# Placebo‐like analgesia via response imagery

**DOI:** 10.1002/ejp.1035

**Published:** 2017-04-19

**Authors:** K.J. Peerdeman, A.I.M. van Laarhoven, D.J.P. Bartels, M.L. Peters, A.W.M. Evers

**Affiliations:** ^1^ Unit Health Medical and Neuropsychology Leiden University the Netherlands; ^2^ Leiden Institute for Brain and Cognition Leiden University the Netherlands; ^3^ Department of Psychiatry Leiden University Medical Center the Netherlands; ^4^ Department of Clinical Psychological Science Maastricht University the Netherlands

## Abstract

**Background:**

Placebo effects on pain are reliably observed in the literature. A core mechanism of these effects is response expectancies. Response expectancies can be formed by instructions, prior experiences and observation of others. Whether mental imagery of a response can also induce placebo‐like expectancy effects on pain has not yet been studied systematically.

**Methods:**

In Study 1, 80 healthy participants were randomly allocated to (i) response imagery or (ii) control imagery. In Study 2, 135 healthy participants were randomly allocated to (i) response imagery with a verbal suggestion regarding its effectiveness, (ii) response imagery only, or (iii) no intervention. In both studies, expected and experienced pain during cold pressor tests were measured pre‐ and post‐intervention, along with psychological and physiological measures.

**Results:**

Participants rated pain as less intense after response imagery than after control imagery in Study 1 (*p *=* *0.044, $\eta_{p}^{2}$ = 0.054) and as less intense after response imagery (with or without verbal suggestion) than after no imagery in Study 2 (*p *<* *0.001, $\eta_{p}^{2}$ **=* *0.154). Adding a verbal suggestion did not affect pain (*p *=* *0.068, $\eta_{p}^{2}$ **=* *0.038). The effects of response imagery on experienced pain were mediated by expected pain.

**Conclusions:**

Thus, in line with research on placebo effects, the current findings indicate that response imagery can induce analgesia, via its effects on response expectancies.

**Significance:**

The reported studies extend research on placebo effects by demonstrating that mental imagery of reduced pain can induce placebo‐like expectancy effects on pain.

## 1 Introduction

Placebo effects demonstrate the importance of expectancies in pain treatment. A rapidly accumulating body of research on the mechanisms of placebo effects indicates that merely expecting that a treatment will provide relief (i.e. response expectancies) can cause pain relief, regardless of the presence of active treatment ingredients (Kirsch, 1997; Benedetti, 2014; Peerdeman et al., 2016). The formation of response expectancies is generally understood to occur by instructions (including verbal suggestion), personal experiences (including conditioning processes) and observation of others (i.e. observational learning; Kirsch, 1997; Colloca and Miller, 2011). Placebo‐like expectancy effects (i.e. expectancy effects without administration of a placebo) (Benedetti, 2014) on pain can possibly also be induced via mental imagery, or simulation, of reduced pain. Mental imagery plays a crucial role in thinking about the past, present and future, and patients with chronic pain commonly experience spontaneous pain‐related mental images (Berna et al., 2012; McNorgan, 2012). Importantly, imagery of sensations largely draws on the same physiological processes as the actual experience of these sensations (Kosslyn et al., 2001; McNorgan, 2012; Fardo et al., 2015), suggesting that imagery might have effects comparable to actual experiences. Evidence for the effects of imagery on expectations comes from research in which participants who were instructed to imagine an event gave a higher estimate of the likelihood of that event happening (Carroll, 1978; Gregory et al., 1985). Furthermore, instructed imagery of a best possible future self or health can affect general expectations of future events (Peters et al., 2010; Hanssen et al., 2013; Peerdeman et al., 2015). Imagery exercises that include images of pain relief have frequently been studied and applied in both experimental and clinical settings, and have been found to provide pain relief (Beers and Karoly, 1979; Devine and Spanos, 1990; Kwekkeboom et al., 2008; Fardo et al., 2015; Peerdeman et al., 2016). However, effects on pain are not unfailingly observed (Wells, 1989; Haase et al., 2005; Jacobson, 2006; Danhauer et al., 2007). Moreover, inferences about the working mechanisms are limited due to the designs employed, e.g. imagery during pain, diverse and multifaceted imagery content, combination with verbal suggestion regarding intended effect and lack of expectancy measures. Thus, although the literature suggests that response imagery of reduced pain may induce placebo‐like expectancy effects on pain, systematic research is lacking.

We aimed to assess whether imagery of reduced pain (i.e. response imagery) could induce analgesia. In Study 1, response imagery was compared to control imagery. In Study 2, response imagery was compared to no intervention, and the effects of adding a verbal suggestion regarding the effectiveness of imagery were assessed. Cold pressor tests were used to assess pain pre‐ and post‐intervention. Our primary hypothesis was that participants would experience less pain after response imagery than after control imagery or no intervention. Secondary, we hypothesized that a verbal suggestion would enhance these effects. Furthermore, we explored whether the effects would be mediated by expected pain. We also explored the possible moderating role of psychological characteristics, evaluations of the imagery intervention and effects on psychological and physiological responses, based on previous literature suggesting that these factors may also be involved (e.g. Geers et al., 2010; Flaten et al., 2011; Schedlowski et al., 2015).

## 2 Study 1

The primary aim of Study 1 was to assess the effects of response imagery on pain, as compared to control imagery.

### 2.1 Methods

#### 2.1.1 Participants

In Study 1, 80 healthy adults participated (power analysis based on previous research) (Beers and Karoly, 1979; Devine and Spanos, 1990; Kwekkeboom et al., 2008). Inclusion criteria were age between 18 and 30 years, and fluency in the Dutch language. See Supporting Information Appendix S1 for specific health‐related exclusion criteria.

#### 2.1.2 Procedure

The study protocol was approved by the institute's ethics committee (Commissie Ethiek Psychologie). Testing took place from March to May 2014 at Leiden University, Leiden, the Netherlands. Participants were recruited via advertisements at and around the university. Potential participants were informed about the evocation of pain with a cold pressor test (CPT) and the use of cognitive tasks. Potential participants filled out screening, demographic and psychological characteristics (optimism, neuroticism) questionnaires (online via Qualtrics, Provo, UT, US; approx. 10 min). Eligible participants were invited to the laboratory and asked to refrain from using medication, alcohol or other drugs in the 24 h prior to the test session, to awaken at least 1 h before the test session, and not to smoke or consume caffeine‐containing drinks or a meal in the hour preceding the session. Testing was done by two experimenters to enable blinding of the outcome assessor. At the beginning of the test session, experimenter A obtained written informed consent from all participants. Subsequently, experimenter A obtained the following pre‐intervention measures consecutively: baseline and expected pain, psychological questionnaires (affect, state anxiety, general expectations), physiological measures (5‐min resting for heart rate and skin conductance; saliva sample for cortisol and alpha‐amylase), and experienced pain, heart rate and skin conductance during the first CPT. Experimenter B then supervised the performance of undemanding filler tasks (e.g. Sudoku puzzles) and obtained two saliva samples (10 and 20 min after CPT). Next, experimenter B introduced the imagery exercise matching the condition to which participants had been randomly allocated (*Response imagery condition* or *Control imagery condition*). For details about the randomization and blinding procedure, see Supporting Information Appendix S2. Post‐intervention, experimenter A obtained the following measures consecutively: expected pain, experienced pain, heart rate and skin conductance during the second CPT, psychological questionnaires (affect, state anxiety, general expectations), questions regarding imagery evaluation and saliva samples (10 and 20 min after CPT). The test session was concluded with an oral debriefing. See Supporting Information Fig. S1 for a flow diagram. The total duration of the test session was 1.5 h. All participants completed the study.

#### 2.1.3 Intervention

Participants in the *Response imagery (Imag) condition* were guided in imagining reduced pain during the imagery exercise that took place prior to the second CPT. They were instructed to vividly imagine that they would experience no or hardly any pain when they would hold their dominant hand in the cold water during the second CPT. They were instructed to do so by imagining that they were wearing a glove, which was described as warm and impermeable to water, and as protecting against the pain one could experience from the cold water. To control for the effects of the content of imagery, participants in the *Control imagery (Contr) condition* merely imagined their hand, without any reference to pain or the cold water. They were instructed to vividly imagine their dominant hand by, for example, closely observing the fingers and palm of the hand and attending to the feeling of moving the hand. In both conditions, the imagery exercise was briefly introduced by the experimenter. Subsequently, audio‐recordings of the detailed instructions were presented via a headphone, using E‐prime 2.0 software (Psychology Software Tools, Inc., Sharpsburg, PA, USA). Participants in both conditions first wrote about their image (3 min), after which they mentally imagined it as vividly as possible (3 min), as in previous studies (Peters et al., 2010; Hanssen et al., 2013). The total duration of the imagery exercise was ~12 min in both conditions. Participants did not receive instructions regarding imagery during the CPT.

#### 2.1.4 Imagery evaluation

Participants rated how well they could visualize and concentrate on the image on a visual analogue scale (VAS) ranging from 0 (*not at all*) to 100 (*very well*). Participants rated the valence of their image on a VAS ranging from 0 (*very negative*) to 100 (*very positive*), and how much they thought about the image during the post‐intervention CPT on a VAS ranging from 0 (*not at all*) to 100 (*very much*).

#### 2.1.5 Cold pressor test

Pain was evoked with a cold pressor test (CPT) (Peerdeman et al., 2015). A Styrofoam tank was filled with non‐circulating cold water of which the temperature was regulated and assessed directly prior to commencing the test (3.9 ± 0.1 °C). Participants immersed their dominant hand up to the wrist in the water and were instructed to hold their hand still and refrain from making a fist or touching the walls of the tank. Participants were unaware of the test duration and were instructed to keep their hand in the water until the experimenter gave a signal (after 1 min). During immersion, participants rated pain intensity every 15 s. The mean pain rating was used for analyses.

#### 2.1.6 Expected and experienced pain

Participants verbally rated expected and experienced pain intensity on a numerical rating scale ranging from 0.0 (*no pain at all*) to 10.0 (*worst pain ever experienced*).

#### 2.1.7 Psychological characteristics

The revised Life Orientation Test (LOT‐R) and the neuroticism scale of the revised short version of the Eysenck Personality Questionnaire (EPQ‐RSS) were used to measure optimism and neuroticism, respectively. For details of the questionnaires, see Supporting Information Appendix S3.

#### 2.1.8 Psychological responses

A short version of the Positive and Negative Affect Schedule (PANAS‐PA and PANAS‐NA), a short version of the State‐Trait Anxiety Inventory (STAI‐S) and the questionnaire for Future Expectations (FEX) were used to measure positive and negative affect, state anxiety, and positive and negative general expectations for future events, respectively. The negative affect data (PANAS‐NA) were not analysed due to floor effects and low internal consistency. For details of the questionnaires, see Supporting Information Appendix S3.

#### 2.1.9 Physiological responses

Heart rate (HR) and skin conductance (SC) were measured continuously using a MP150 system and AcqKnowledge software, version 4.3.1 (BIOPAC Systems Inc., Goleta, CA, USA) according to standard procedures. Saliva samples were collected with cotton swabs (Salivette, Sarstedt, Nümbrecht, Germany) for assessments of cortisol and alpha‐amylase. The samples were processed according to standard procedures. For more details, see Supporting Information Appendix S4.

#### 2.1.10 Statistical analyses

All data were analysed using IBM SPSS Statistics version 21 (IBM Corporation, Armonk, NY, USA), with a two‐tailed significance level of α = 0.05. Descriptives are reported as means and standard deviations (*M *± SD). The effects of response imagery on post‐intervention experienced pain (primary outcome), expected pain, positive affect, state anxiety, general expectations, heart rate, skin conductance, cortisol and alpha‐amylase were analysed with separate univariate analyses of covariance (ANCOVAs; determined *a priori*) (Van Breukelen, 2006). Imagery was the independent variable (*Imag* vs. *Contr condition),* the post‐intervention measures were dependent variables, and the corresponding pre‐intervention measures and stratification variables (sex and time of day) were covariates. The possible mediating role of expected pain in the effect of response imagery on experienced pain was explored using an ordinary least squares regression approach. To determine mediation, bias‐corrected 95% confidence intervals were calculated for the indirect effect using 1000 bootstrapping samples via the Process SPSS macro (Hayes, 2013). Mediation was confirmed if the confidence interval did not include zero (Hayes, 2013). The pre‐intervention measures and stratification variables were included as covariates in the mediation model. The possible moderating influence of trait optimism and neuroticism on the effects of imagery on experienced pain was explored via separate multiple regression analyses. Moderation was confirmed if the interaction of the psychological characteristic in question with the imagery conditions was significant in the regression model in which the psychological characteristic, imagery conditions, pre‐intervention experienced pain, stratification variables and the interaction were simultaneously entered as predictors of post‐intervention experienced pain. Imagery evaluations were compared between conditions with separate univariate ANCOVAs, with the stratification variables as covariates. Means and standard deviations for all measures are reported in Supporting Information Table S1.

Additional *post hoc* correlation analyses (associations of post‐intervention experienced pain with post‐intervention imagery evaluation, psychological responses and physiological responses) and sensitivity analyses (in case of violation of the assumptions of statistical tests and doubts about inclusion) are described in Supporting Information Appendix S5 and reported in Supporting Information Appendix S6. In Supporting Information Appendix S5, also detailed information on missing data is reported.

### 2.2 Results

#### 2.2.1 Participants

Thirty‐nine participants were allocated to the *Imag condition* (age 20.8 ± 2.4, 67% women) and 41 to the *Contr condition* (age 21.1 ± 2.0, 66% women). Participants in both conditions reported low baseline pain (0.1 ± 0.2 and 0.1 ± 0.4, respectively). There were no significant differences between the conditions in age, sex and baseline pain.

#### 2.2.2 Effects on experienced pain

**Figure 1:**
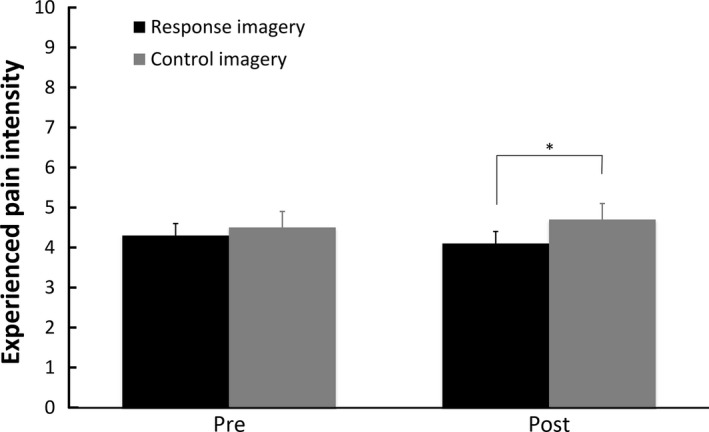
Means and standard errors of experienced pain intensity ratings for the pre‐ and post‐intervention cold pressor tests per condition in Study 1. *p <* *0.05; **p <* *0.01; ***p <* *0.001.

In line with the primary hypothesis, mean ratings of experienced pain during the post‐intervention CPT (see Fig. 1) were significantly lower after response imagery than after control imagery [*F*(1, 74) = 4.192, *p* = 0.044, $\eta_{p}^{2}$ = 0.054].

#### 2.2.3 Mediation by expectancy

**Figure 2:**
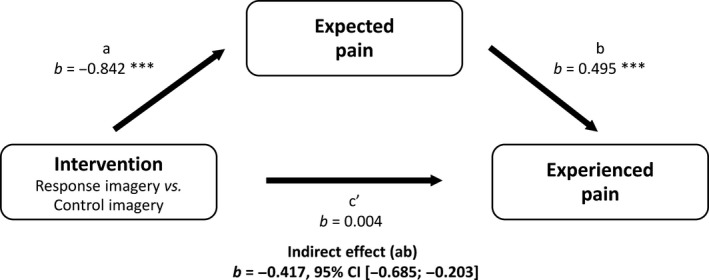
Mediation of effect of response imagery on experienced pain by expected pain, Study 1. *p <* *0.05; **p <* *0.01; ***p <* *0.001.

Expected pain ratings were significantly lower after response imagery than after control imagery [*F*(1, 75) = 4.030, *p* = 0.048, $\eta_{p}^{2}$ = 0.051]. Moreover, the effect of response imagery on experienced pain was mediated by expected pain [*b* = −0.417, 95% CI (−0.685; −0.203)]. See Fig. 2 for the coefficients of all paths in the mediation model.

#### 2.2.4 Moderation by psychological characteristics

The effect of imagery on experienced pain was not significantly moderated by optimism or neuroticism, as indicated by non‐significant interactions of the imagery conditions with the LOT‐R (*β *= 0.131, *t *= 0.815, *p *=* *0.418) and EPQ‐RSS scores (*β *= 0.046, *t *=* *0.280, *p *=* *0.780).

#### 2.2.5 Imagery evaluation

There were no significant differences between the response imagery and control condition in how participants rated the quality of the visualization [*F*(1, 75) = 0.369, *p *=* *0.546, $\eta_{p}^{2}$ = 0.005] or their concentration on the image [*F*(1, 75) = 0.655, *p *=* *0.421, $\eta_{p}^{2}$ = 0.009]. Participants in the *Imag condition* rated the image as significantly more positive [*F*(1, 75) = 5.542, *p *=* *0.021, $\eta_{p}^{2}$ = 0.069] and thought more about the image during the post‐intervention CPT [*F*(1, 75) = 42.157, *p *<* *0.001, $\eta_{p}^{2}$ = 0.360] than participants in the *Contr condition*.

#### 2.2.6 Effects on psychological responses

There were no significant effects of response imagery on positive affect [PANAS‐PA; *F*(1, 75) = 0.637, *p* = 0.427, $\eta_{p}^{2}$ = 0.008], state anxiety [STAI‐S; *F*(1, 75) = 0.009, *p* = 0.924, $\eta_{p}^{2}$ < 0.001], general positive expectations [FEXpos; *F*(1, 75) = 3.718, *p *=* *0.058, $\eta_{p}^{2}$ = 0.047] or general negative expectations [FEXneg; *F*(1, 75) = 3.297, *p* = 0.073, $\eta_{p}^{2}$ = 0.042].

#### 2.2.7 Effects on physiological responses

There was no significant effect of response imagery on heart rate during the post‐intervention CPT [*F*(1, 73) = 1.461, *p* = 0.231, $\eta_{p}^{2}$ = 0.020]. Excluding the data of one participant who had a very irregular heart rate did not significantly affect the results [*F*(1, 72) = 1.368, *p* = 0.246, $\eta_{p}^{2}$ = 0.019]. There were also no significant effects of response imagery on skin conductance during the post‐intervention CPT [*F*(1, 74) = 0.005, *p* = 0.943, $\eta_{p}^{2}$ < 0.001], cortisol and alpha‐amylase 10 min after the post‐intervention CPT [*F*(1, 74) = 0.131, *p* = 0.718, $\eta_{p}^{2}$ = 0.002 and *F*(1, 73) = 0.069, *p* = 0.794, $\eta_{p}^{2}$ = 0.001, respectively], or cortisol and alpha‐amylase 20 min after the post‐intervention CPT [*F*(1, 75) = 1.936, *p* = 0.168, $\eta_{p}^{2}$ = 0.025 and *F*(1, 74) = 2.026, *p* = 0.159, $\eta_{p}^{2}$ = 0.027, respectively].

## 3 Study 2

The aim of Study 2 was to replicate and extend the findings of Study 1. We again assessed the effect of response imagery on pain, but in this study we used a different control condition. While participants in the control condition of Study 1 imagined their hand, to assess the influence of the specific contents of imagery rather than the process of imagery, participants in the control condition of Study 2 did nothing, to assess the effects of the mere passage of time (natural history), and to thereby allow for a comparison that is more representative of clinical practice. An additional reason for using a different control condition in Study 2, was that we were concerned that the image used in the control condition of Study 1 might also affect pain; merely imagining one's hand, which was previously immersed in the cold water, might reduce pain via mindfulness‐like processes (Reiner et al., 2013), or might alternatively increase pain by enhancing awareness of the pain (Bantick et al., 2002). Secondary, we aimed to assess whether the effects of response imagery on pain could be enhanced by adding a verbal suggestion. We therefore added a third condition, in which the response imagery exercise was preceded by a verbal suggestion of its effectiveness. We did not assess salivary cortisol and alpha‐amylase in Study 2, since these measures were not sensitive to the intervention in Study 1.

### 3.1 Methods

#### 3.1.1 Participants

In Study 2, 135 healthy adults participated (power analysis based on Study 1 and previous research) (Beers and Karoly, 1979; Devine and Spanos, 1990; Kwekkeboom et al., 2008). Inclusion and exclusion criteria were the same as in Study 1, except that current use of all types of medication was now an exclusion criterion. In addition, people could not participate in Study 2 if they had participated in Study 1.

#### 3.1.2 Procedure

Following approval by the institute's ethics committee, testing took place from October 2014 to February 2015 at Leiden University, Leiden, the Netherlands. The general procedure was the same as in Study 1, with the exception of the specific intervention given, the omission of salivary cortisol and alpha‐amylase assessments (and consequently omission of instructions regarding waking time and eating prior to participation), and the addition of the following measures: a pain catastrophizing questionnaire was administered with the pre‐test session questionnaires; an extra assessment of expected pain was done after the pre‐intervention CPT to obtain a pre‐intervention expectancy score that was informed by the actual pain induced by a CPT; and pain anxiety was assessed directly following each expected pain assessment. See Supporting Information Fig. S2 for a flow diagram.

#### 3.1.3 Intervention

As in Study 1, participants in the *Response imagery (Imag) condition* imagined reduced pain using the image of a glove during the imagery exercise that took place prior to the second CPT. The imagery instructions were largely the same, but the phrasing of the instructions was slightly improved (e.g. ‘Imagine that you can fully relax your hand and that you feel hardly or no pain…’ in Study 1 vs. ‘Imagine that you feel hardly or no pain […]. You will be able to fully relax your hand’ in Study 2). Participants first wrote about their image (3 min), after which they imagined it as vividly as possible (2 min). Participants in the *Response imagery with verbal suggestion (Imag+VS) condition* did the same response imagery exercise, but this was preceded by a verbal suggestion that described the effectiveness of the exercise, by stating, among other things, ‘we know from previous scientific research that this imagery exercise is effective’ and ‘almost everyone experiences much less pain due to this exercise’. Participants in the *No treatment control (NT Contr) condition* waited, while reading a magazine, for the same duration as the imagery exercise (approx. ~12 min).

#### 3.1.4 Measures

In addition to the measures used in Study 1, two additional measures were used. The pain catastrophizing scale (PCS) was used to measure pain catastrophizing. A numerical rating scale (0.0–10.0) was used to assess pain anxiety, but the data were not analysed due to floor effects. For details of the questionnaires, see Supporting Information Methods S3.

#### 3.1.5 Statistical analyses

The same procedures and analyses were used as in Study 1 to assess the effects of response imagery and of adding a verbal suggestion on pain (primary and secondary analyses, respectively), and to explore the possible mediation by expected pain, the possible moderating role of psychological characteristics, differences in imagery evaluation, and the effects on the other self‐reported and physiological measures. To assess the effects of response imagery, the *Imag condition* and the *Imag+VS condition* were taken together and compared to the *NT Contr condition* in all analyses. We determined to pool the imagery conditions *a priori*, to maximize power and readability. However, for the primary outcome, we also reported *post hoc* comparisons of the individual imagery conditions with the control condition for completeness. To assess the effects of adding a verbal suggestion to the response imagery exercise, the *Imag condition* and the *Imag+VS condition* were compared with each other in all analyses. Means and standard deviations for all measures are reported in Supporting Information Table S2.

As in Study 1, additional *post hoc* correlation analyses and sensitivity analyses are described in Supporting Information Methods S5 and reported in Supporting Information Appendix S6. In Supporting Information Methods S5 also detailed information on missing data is reported.

## 4 Results

### 4.1 Participants

Forty‐seven participants were allocated to the *Imag+VS condition* (age 21.8 ± 2.7, 85% women), 45 to the *Imag condition* (age 20.6 ± 1.8, 82% women) and 43 to the *NT Contr condition* (age 21.1 ± 2.9, 81% women). Participants in all conditions reported low baseline pain (0.0 ± 0.2; 0.1 ± 0.3; 0.1 ± 0.3, respectively). There were no significant differences between the conditions in age, sex and baseline pain, except for significantly older age in the *Imag+VS condition* than in the *Imag condition* [*F*(1, 89) = 7.254, *p* = 0.008, $\eta_{p}^{2}$ = 0.075].

### 4.2 Effects on experienced pain

**Figure 3:**
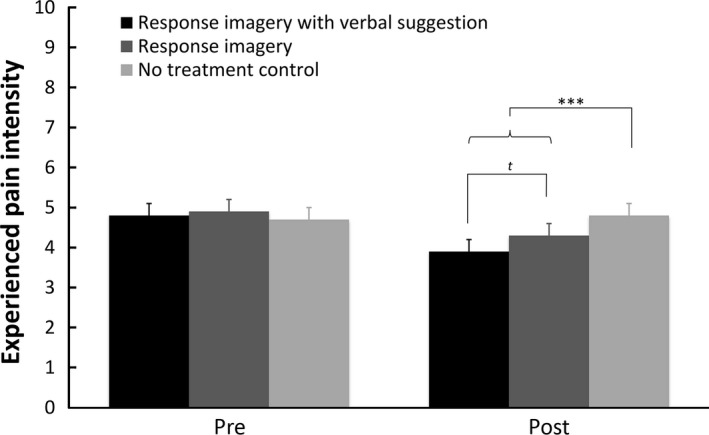
Means and standard errors of experienced pain intensity ratings for the pre‐ and post‐intervention cold pressor tests per condition in Study 2. ^*t*^ *p* < 0.10; *p <* *0.05; **p <* *0.01; ***p <* *0.001.

In line with the primary hypothesis, mean ratings of experienced pain during the post‐intervention CPT (see Fig. 3) were significantly lower after response imagery (regardless of verbal suggestion) than after no intervention [*F*(1, 130) = 23.613, *p *<* *0.001, $\eta_{p}^{2}$ = 0.154]. Further *post hoc* comparisons of the individual imagery conditions with the control condition, showed this difference both when a verbal suggestion was added to response imagery [*F*(1, 86) = 24.896, *p *<* *0.001, $\eta_{p}^{2}$ = 0.225] and when response imagery was given alone [*F*(1, 83) = 12.420, *p* = 0.001, $\eta_{p}^{2}$ = 0.130]. In contrast to the secondary hypothesis, adding a verbal suggestion did not affect experienced pain ratings, although a trend was observed [*F*(1, 87) = 3.423, *p* = 0.068, $\eta_{p}^{2}$ = 0.038].

### 4.3 Mediation by expectancy

**Figure 4:**
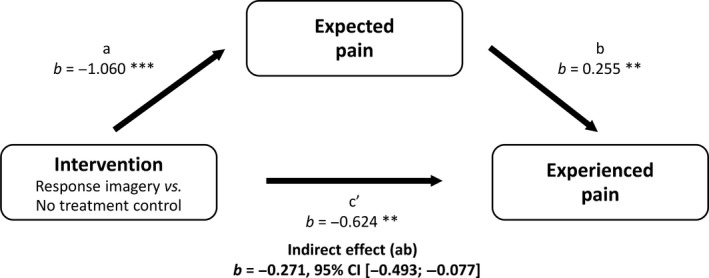
Mediation of effect of response imagery on experienced pain by expected pain, Study 2. *p <* *0.05; **p <* *0.01; ***p <* *0.001.

**Figure 5:**
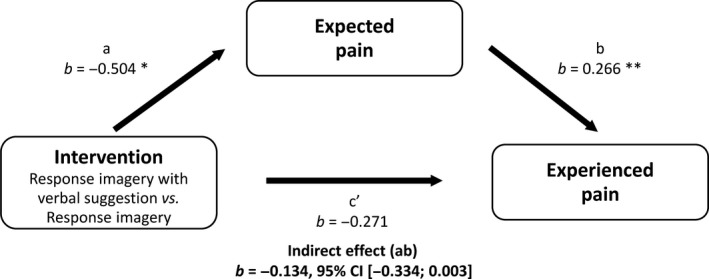
Mediation of effect of verbal suggestion about response imagery on experienced pain by expected pain, Study 2. *p <* *0.05; **p <* *0.01; ***p <* *0.001.

Expected pain ratings were significantly lower after response imagery than after no intervention [*F*(1, 129) = 30.908, *p *<* *0.001, $\eta_{p}^{2}$ = 0.193]. Similarly, adding a verbal suggestion to the imagery exercise led to significantly lower expected pain intensity ratings in the *Imag+VS condition* than in the *Imag condition* [*F*(1, 86) = 4.981, *p* = 0.028, $\eta_{p}^{2}$ = 0.055]. The effect of response imagery on experienced pain was mediated by expected pain [*b* = −0.271, 95% CI (−0.493; −0.077)], while the effect of adding a verbal suggestion on experienced pain was not mediated by expected pain [*b* = −0.134, 95% CI (−0.334; 0.003)]. See Figs. 4 and 5 for the coefficients of all paths in the mediation models.

### 4.4 Moderation by psychological characteristics

The effect of response imagery on experienced pain was not moderated by optimism, neuroticism or pain catastrophizing, as indicated by non‐significant interactions of the LOT‐R (β = −0.004, *t* = −0. 056, *p* = 0.955), EPQ‐RSS (β = −0.032, *t* = −0.404, *p* = 0.687), and PCS scores (β = 0.087, *t* = 1.125, *p* = 0.263) with the imagery conditions. Similarly, the effect of adding a verbal suggestion on experienced pain was not significantly moderated by optimism or pain catastrophizing (β = −0.064, *t* = −0.407, *p* = 0.685; and β = 0.126, *t* = 0.786, *p* = 0.434, respectively). The effect of adding a verbal suggestion on experienced pain did appear to be moderated by neuroticism (β = 0.326, *t* = 2.024, *p* = 0.046). Follow‐up analyses indicated that an effect of verbal suggestion was only present for participants who scored high on neuroticism (1 SD above the mean; *b* = 0.740, *t* = 2.554, *p* = 0.012).

### 4.5 Imagery evaluation

There were no significant differences between the imagery conditions in vividness of the image [*F*(1, 87) = 0.426, *p* = 0.515, $\eta_{p}^{2}$ = 0.005], concentration on the image [*F*(1, 87) = 0.068, *p* = 0.796, $\eta_{p}^{2}$ = 0.001], valence of the image [*F*(1, 87) = 0.811, *p* = 0.370, $\eta_{p}^{2}$ = 0.009] and thinking about the image during the post‐intervention CPT [*F*(1, 87) = 2.580, *p* = 0.112, $\eta_{p}^{2}$ = 0.029].

### 4.6 Effects on psychological responses

Participants in the response imagery conditions reported significantly higher general positive expectations [FEXpos; *F*(1, 130) = 5.261, *p* = 0.023, $\eta_{p}^{2}$ = 0.039] than participants in the *NT Contr condition*. There were no significant effects of response imagery on positive affect [PANAS‐PA; *F*(1, 130) = 3.896, *p* = 0.051, $\eta_{p}^{2}$ = 0.029], state anxiety [STAI‐S; *F*(1, 130) = 0.152, *p* = 0.697, $\eta_{p}^{2}$ = 0.001] or general negative expectations [FEXneg; *F*(1, 130) = 0.130, *p* = 0.719, $\eta_{p}^{2}$ = 0.001]. Adding a verbal suggestion to the response imagery exercise did not significantly influence positive affect [PANAS‐PA; *F*(1, 87) = 0.003, *p* = 0.956, $\eta_{p}^{2}$ < 0.001], state anxiety [STAI‐S; *F*(1, 87) = 2.439, *p* = 0.122, $\eta_{p}^{2}$ = 0.027], general positive expectations [FEXpos; *F*(1,87) = 0.330, *p* = 0.567, $\eta_{p}^{2}$ = 0.004] or general negative expectations [FEXneg; *F*(1, 87) = 1.028, *p* = 0.313, $\eta_{p}^{2}$ = 0.012].

### 4.7 Effects on physiological responses

There was no significant effect of response imagery on heart rate [*F*(1, 128) = 3.885, *p* = 0.051, $\eta_{p}^{2}$ = 0.029] or skin conductance [*F*(1, 128) = 3.261, *p* = 0.073, $\eta_{p}^{2}$ = 0.025] during the post‐intervention CPT. Adding a verbal suggestion did not significantly influence heart rate [*F*(1, 87) = 0.367, *p* = 0.546, $\eta_{p}^{2}$ = 0.004] or skin conductance [*F*(1, 87) = 2.490, *p* = 0.118, $\eta_{p}^{2}$ = 0.028].

## 5 Discussion

In two experimental studies, response imagery, i.e. imagery of reduced pain, was found to induce analgesia via its effects on response expectancies, with statistically small to medium effects in Study 1 and large effects in Study 2. An additional verbal suggestion regarding the effectiveness of imagery did not significantly affect pain. These findings suggest that response imagery can affect future pain responses and can be viewed as a possible technique for inducing placebo‐like effects (i.e. expectancy effects without administration of a placebo) (Benedetti, 2014).

The current findings extend previous research on the mechanisms of placebo effects by showing that placebo‐like expectancy effects on pain can be induced not only by instructions, direct experience and observation of other people (Kirsch, 1997; Colloca and Miller, 2011), but also by mental imagery of a response (i.e. simulated experience). This is consistent with response expectancy theory (Kirsch, 1997) and neurobiological findings indicating that brain activation is similar during actual and imagined sensations (McNorgan, 2012; Fardo et al., 2015). The observed effects of response imagery on pain support our primary hypothesis and are in line with previous studies that demonstrate that imagery exercises including images of pain reduction can reduce experimentally evoked pain as well as acute and chronic clinical pain (although effect sizes are heterogeneous) (Beers and Karoly, 1979; Devine and Spanos, 1990; Kwekkeboom et al., 2008; Fardo et al., 2015; Peerdeman et al., 2016). The effects in Study 2 are comparable in size with placebo effects in healthy controls and patients with pain (Vase et al., 2002, 2009; Peerdeman et al., 2016). By instructing participants to imagine reduced pain prior to the pain experience (rather than during as is common in clinical interventions) (Van Kuiken, 2004), and by including a measure of expected pain, we found, for the first time, evidence that the effects of response imagery on experienced pain can be mediated by expected pain. Hereby, we further increase the knowledge on the working mechanisms of imagery. These findings suggest that response imagery might provide an additional manner to harness placebo‐like expectancy effects, without placebo administration or deception.

In addition to the effect of imagery, we studied the effects of providing a positive verbal suggestion regarding the effectiveness of the response imagery intervention. Such a verbal suggestion corresponds with procedures in previous research and in clinical practice, where imagery interventions are generally introduced with information regarding the intended and/or expected outcomes. Contrary to our secondary hypothesis, participants who had received the verbal suggestion did not experience less pain than participants who only received the imagery instructions, although a statistical trend in this direction was observed and participants expected less pain. Possibly, a ceiling effect occurred where verbal suggestion could not elicit a significant effect on pain above that of response imagery alone. Our finding is partially consistent with a large body of research demonstrating the successful induction of placebo effects by verbal suggestion (Vase et al., 2002; Peerdeman et al., 2016). Future research might elucidate whether adding a verbal suggestion can indeed enhance the effects of response imagery, taking into account factors such as the specific phrasing of the suggestion, and perhaps providing a suggestion more frequently to enhance encoding and effects.

Expectancies are generally seen as the core mechanism of placebo effects, but other psychological working mechanisms could also be considered when trying to explain the effect of response imagery on pain. For example, negative emotions have been suggested to mediate the effects of placebos on pain (Flaten et al., 2011) [although previous imagery and placebo studies had equivocal results (Staats et al., 1998; Aslaksen and Flaten, 2008; Peerdeman et al., 2016)] and attention processes might also partially explain effects of response imagery on pain (Eccleston and Crombez, 1999; Bantick et al., 2002) (but see Buhle et al., 2012). Exploratory analyses of the current data showed that general expectancies, positive affect and state anxiety are unlikely to have played a substantial role in bringing about the effects of response expectancy. The involvement of attention processes during both the imagery exercise and the CPT cannot be fully excluded. For example, our findings indicate that participants in the response imagery conditions thought about the image during the post‐intervention CPT, even though they had not received instructions to do so, which could have distracted them from the evoked pain. Future research might investigate the mechanisms further, e.g. by including other measures and/or directly comparing the mediation by response expectancies with mediation by emotions, attention, and general expectations.

In the current studies, our exploratory analyses did not indicate reliable effects of response imagery on autonomic and endocrine responses, even though response imagery was found to affect pain. This could give rise to concerns about the influence of demand characteristics. However, since previous studies did find the effects of pain‐focused imagery on pain and placebo analgesia to be associated with corresponding effects on the autonomic nervous system and with the activation of brain responses that are known to be involved in pain experiences and expectancies (Kosslyn et al., 2001; McNorgan, 2012; Atlas and Wager, 2014; Fardo et al., 2015; Schedlowski et al., 2015), it is likely that the autonomic nervous system was also involved in the effects of response imagery on pain in the current studies. The existing evidence for the involvement of the endocrine system is less convincing (Flaten et al., 2006; Schmid et al., 2015). Methodological factors are likely to have affected our results regarding physiological responses. It is possible that effects on physiological responses were obscured by large inter‐individual variability and lower sensitivity of the responses; we observed large variability of particularly the alpha‐amylase responses, and heart rate was only slightly affected by the CPTs, even though the CPTs evoked moderate pain (comparable to previous studies; van Laarhoven et al., 2010; Peerdeman et al., 2015). Furthermore, the cortisol and alpha‐amylase responses appeared to be affected by the circadian rhythm. Future studies using more sensitive physiological responses and/or measurement techniques, more rigorous controlling of circadian rhythm (Kirschbaum and Hellhammer, 2000; Rohleder and Nater, 2009), larger sample sizes and possibly also other types of experimental as well as clinical pain, might allow more definite conclusions regarding the physiological correlates of the effects of response imagery. Furthermore, additional self‐report measures, such as social desirability questionnaires, may also provide more insight into the possible influence of demand characteristics, although previous research using such measures did not find this to be a significant factor (Morton et al., 2009; van Laarhoven et al., 2011).

Finally, individual differences in psychological characteristics might determine the effectiveness of response imagery. Although some previous studies have found optimism, neuroticism and pain catastrophizing to be associated with the analgesic effects of imagery or placebo‐related expectancy inductions (Geers et al., 2010; Hanssen et al., 2013; Darragh et al., 2014), several other studies did not find any such association (van Laarhoven et al., 2011; Hanssen et al., 2014; Peerdeman et al., 2015). In the current studies, we found no evidence for the moderation of the effects of imagery on pain by optimism or pain catastrophizing, but some indications that neuroticism might play a role in the effects of verbal suggestion. Future research might further investigate the determinants of response imagery and placebo effects, by studying not only individual differences in psychological characteristics, but also in pre‐existing expectancies (e.g. due to previous experiences) and different types of pain (e.g. acute vs. chronic pain) (Horing et al., 2014; Peerdeman et al., 2016). Furthermore, participants received standardized and detailed instructions for the imagery exercise. An advantage was that all participants could imagine a concrete image of an otherwise abstract concept. This is especially helpful for people who otherwise have trouble constructing an image themselves (Kwekkeboom et al., 2008). Moreover, as postulated in the simulation heuristic (Tversky and Kahneman, 1973) and observed in several studies (Brown et al., 2002; Raune et al., 2005), the ease with which a mental image can be constructed has been associated with its effects on individuals' expectations of events. Many chronic pain patients, however, experience spontaneous, highly individual, pain‐related images (Berna et al., 2012), and it might be beneficial for them to form their own personal images of pain reduction instead of visualizing a standard image. Indeed, one study found the rescripting of pain patients' most distressing pain image to a preferred, self‐generated, image to be very beneficial (Philips and Samson, 2012).

In conclusion, the current findings indicate that a brief response imagery intervention can induce placebo‐like expectancy effects on pain. If these findings can be replicated and extended, in both healthy and clinical samples, response imagery could ultimately be implemented in clinical practice to optimize expectations and thereby improve the effectiveness of standard pain treatments.

## Author contributions

All authors discussed the results and commented on the manuscript. All authors contributed to the conception and design of the studies. K.P. collected and processed the data. K.P. and Av.L. conducted data analyses. All authors contributed to the interpretation of the results. All authors contributed to the manuscript and have read and approved the manuscript.

## Supporting information


**Figure S1.** Flow diagram showing the experimental procedures of Study 1 in chronological order.
**Figure S2.** Flow diagram showing the experimental procedures of Study 2 in chronological order.
**Table S1.** Means and standard deviations for all measures in Study 1.
**Table S2.** Means and standard deviations for all measures in Study 2.
**Appendix S1.** Health‐related exclusion criteria.
**Appendix S2.** Randomization & blinding procedure.
**Appendix S3.** Psychological characteristics & responses.
**Appendix S4.** Physiological responses.
**Appendix S5.** Methods additional analyses.
**Appendix S6.** Results additional analyses.
