# Supplementary material for: Placebo‐like analgesia via response imagery
Source: Eur J Pain. 2017 Apr 19;21(8):1366–77. doi: 10.1002/ejp.1035 (PMC5573948; doi:10.1002/ejp.1035)
Supplement: Supplementary file 1 — Figure S1. Flow diagram showing the experimental procedures of Study 1 in chronological order. Figure S2. Flow diagram showing the experimental procedures of Study 2 in chronological order. Table S1. Means and standard deviations for all measures in Study 1. Table S2. Means and standard deviations for all measures in Study 2. Appendix S1. Health‐related exclusion criteria. Appendix S2. Randomization & blinding procedure. Appendix S3. Psychological characteristics & responses. Appendix S4. Physiological responses. Appendix S5. Methods additional analyses. Appendix S6. Results additional analyses. [file EJP-21-1366-s001.docx]

# Supporting information

## Appendix S1: Health-related exclusion criteria

In Study 1, health-related exclusion criteria were severe physical or psychological morbidity (e.g., heart disease or DSM-IV psychiatric disorders) that would adversely affect participation, current chronic (≥ 6 months) pain complaints, Raynaud’s phenomenon, extensive injuries to the hand to be immersed, current medication use (specifically, analgesics, anti-inflammatory drugs, antihistamines, antibiotics, beta-blockers, or other medications that influence heart rate), use of pacemaker, and pregnancy.

In Study 2, exclusion criteria were the same as in Study 1, except that current use of all types of medication was now an exclusion criterion.

## Appendix S2: Randomization & blinding procedure

In Study 1, the randomization sequence was generated by an independent researcher with an online random number generator (www.randomization.com; stratified by sex and time of day [morning vs. afternoon], with a 1:1 allocation using blocks of 4 and 6). Allocation was concealed by using sequentially numbered, opaque, sealed envelopes. As indicated, testing was done by two experimenters to enable blinding of the outcome assessor. Experimenter A, who conducted the measurements, was unaware of allocation throughout the test session. Experimenter B, who led the intervention (including the preceding filler tasks), was unaware of allocation until intervention onset. The experimenters did not communicate about any aspects of the procedure that could lead to unblinding. To maximize blinding of the participants, they were not informed about the different experimental conditions until debriefing.

In Study 2, the randomization sequence was generated according to the same procedures as used for Study 2, except for stratification by sex only (i.e., omission of stratification on time of day), and 1:1:1 allocation using blocks of 3, 6, and 9. The blinding procedures were the same as in Study 1.

## Appendix S3: Psychological characteristics & responses

Dispositional optimism. In Study 1 and 2, the revised Life Orientation Test (LOT-R) (Scheier et al., 1994; Peters et al., 2010) was used to measure dispositional optimism (3 positive, 3 negative, and 4 filler items, 5-point Likert scale). The total score ranges from 0 to 24.

Neuroticism. In Study 1 and 2, the neuroticism scale of the revised short version of the Eysenck Personality Questionnaire (EPQ-RSS) (Sanderman et al., 1995) was used to measure neuroticism (12 items, dichotomous (yes/no) scale). The total score ranges from 0 to 12.

Pain catastrophizing. In Study 2, the Pain Catastrophizing Scale (PCS) (Sullivan et al., 1995) was used to measure pain catastrophizing (13 statements, 5-point Likert scale). The total score ranges from 0 to 52.

Affect. In Study 1 and 2, a short version of the Positive and Negative Affect Schedule (PANAS) (Kercher, 1992; Peeters et al., 1996) was used to measure positive and negative affect (5 positive items, i.e., PANAS-PA, and 5 negative items, i.e., PANAS-NA, 5-point Likert scale). The PANAS-PA and PANAS-NA scores both range from 5 to 25.

State anxiety. In Study 1 and 2, a short version of the State-Trait Anxiety Inventory, State version (STAI-S) (van der Ploeg, 1982; Marteau and Bekker, 1992) was used to measure state anxiety (3 negative and 3 positive statements, 4-point Likert scale). The total score ranges from 20 to 80 (after multiplication by 3.33 for comparability with the full scale).

General expectations. In Study 1 and 2, the questionnaire for Future Expectations (FEX) (Hanssen et al., 2013) was used to measure positive and negative general expectations for future events (10 positive future events, i.e., FEXpos, and 10 negative future events, i.e., FEXneg, 7-point Likert scale). The FEXpos and FEXneg both range from 10-70.

Pain anxiety. In Study 2, a numerical rating scale ranging from 0.0 (*not* *anxious* *at all*) to 10.0 (*most anxious* *ever experienced)* was used to assess pain anxiety, i.e., anxiety regarding the coming CPT.

Psychometric properties questionnaires. Cronbach’s alpha of the questionnaires was satisfactory for almost all questionnaires at pre- and post-intervention, except for the PANAS-NA (Study 1 and 2) and the FEXpos (Study 1) (see the table below). The PANAS-NA data were not analyzed due to the low internal consistency and floor effects (Study 1: post-intervention, 85% of participants reported minimum score; Study 2: post-intervention, 77% of participants reported the minimum score). For the FEXpos a satisfactory Cronbach’s alpha for this scale was obtained after excluding item 4 in Study 1 (α = .68 to .71). Last, the pain anxiety data were not analyzed due to floor effects (post-intervention, 47% of participants reported no pain anxiety).

| Cronbach’s alpha | | |
| --- | --- | --- |
|  | Study 1 | Study 2 |
| LOT-R | .77 | .69 |
| EPQ-RSS | .80 | .72 |
| PCS | n.a. | .89 |
| PANAS-PA |  |  |
| pre-intervention | .70 | .71 |
| post-intervention | .76 | .73 |
| PANAS-NA |  |  |
| pre-intervention | .67 | .65 |
| post-intervention | .45 | .49 |
| STAI-S |  |  |
| pre-intervention | .73 | .82 |
| post-intervention | .66 | .74 |
| FEXpos |  |  |
| pre-intervention | .56 | .84 |
| post-intervention | .51 | .87 |
| FEXneg |  |  |
| pre-intervention | .68 | .81 |
| post-intervention | .68 | .84 |

Appendix **S4: Physiological responses**

Heart rate and skin conductance. In Study 1 and 2 heart rate (HR) and skin conductance (SC) were measured continuously using a MP150 system and AcqKnowledge software, version 4.3.1 (BIOPAC Systems Inc., Goleta, CA, USA). For HR measurements, a disposable electrode (Kendall 200 Foam Electrode, Covidien, Mansfield, MA, USA) was placed on the sternum and another on the left lower rib, after abrading the skin. Electrocardiography (ECG) signals were recorded with an ECG100C amplifier (1000 Hz, gain 1000, 0.5 Hz high pass filter). For SC measurements, disposable Ag/AgCl electrodes (EL507-10, BIOPAC Systems Inc., Goleta, CA, USA) were placed on the medial phalanges of the index and middle finger of the non-dominant hand, after cleaning the skin with water. SC level (µS) was recorded with a GSR100C amplifier (1000 Hz, gain 5 µmho/V, 10.0 Hz low pass filter). Inspection of the ECG and SC data, HR calculation, and calculation of the mean HR and SC levels during baseline and the CPTs was conducted in MATLAB (version R2012b, the MathWorks, Inc., Natick, Ma, USA). Epochs were marked using triggers via E-prime 2.0 software.

Cortisol and alpha-amylase. In Study 1, saliva samples were collected with cotton swabs (Salivette, Sarstedt, Nümbrecht, Germany) for assessments of cortisol and alpha-amylase (Kirschbaum and Hellhammer, 1994; Kirschbaum and Hellhammer, 2000; Rohleder and Nater, 2009). The samples were initially stored at -20°C. After the samples had been thawed and centrifuged, 0.5 ml aliquots were stored at -80°C until biochemical analyses at the Department of Clinical Chemistry and Laboratory Medicine of the Leiden University Medical Center, Leiden, the Netherlands. Cortisol (nmol/L) was measured with a Modular P800 (Roche, Mannheim, Germany). Alpha-amylase (U/L) was measured, in a 25μL sample diluted with 2475 μL saline, with an Integra 800 (Roche, Mannheim, Germany).

## Appendix S5: Additional analyses

Correlation of experienced pain with other outcomes Study 1 and 2. Post hoc partial correlation analyses were conducted in the imagery condition(s) to explore the association of post-intervention experienced pain with post-intervention imagery evaluation, psychological responses, and physiological responses; in these analyses we controlled for the matching pre-intervention measures (e.g., pre-intervention experienced pain and positive affect when assessing the association between post-intervention experienced pain and positive affect) and the stratification variable(s).

Sensitivity analyses Study 1 and 2. In case the assumptions of statistical tests (e.g., of normality) were violated, sensitivity analyses were conducted by 1) calculating bias-corrected 95% confidence intervals around the relevant parameter using 1000 bootstrapping samples, 2) transforming the data (e.g., log transformation), and/or 3) reanalyzing the data without outliers.

Sensitivity analyses were also conducted to assess the influence of excluding the data of participants who had inadvertently used medication that might have affected their responses (Study 1) or who had indicated complaints that could be characterized as Raynaud’s phenomenon (Study 2).

Missing data Study 1. Some data were missing due to practical and/or technical issues: experienced pain ratings (pre-intervention, *n* = 1), heart rate (pre-intervention, *n* = 1; full data, *n* = 1), and skin conductance levels (pre-intervention, *n* = 1), cortisol and alpha-amylase (10 min after first CPT, *n* = 1), and imagery evaluation data (*n* = 1). For one participant alpha-amylase values were unreliably low and therefore not analyzed. All participants completed both cold pressor tests.

Missing data Study 2. One participant withdrew from participation during the pre-intervention CPT due to illness unrelated to the study. Experienced pain ratings were partially missing for four participants (1 in the *Imag+VS condition*, i.e., 2%, 3 in the *Imag condition*, i.e., 7%) who ended the pre-intervention CPT prematurely (< 1 min), and one participant (in the *Imag+VS condition*) who ended the post-intervention CPT prematurely due to pain intensity. These missing ratings were replaced using the last observation carried forward method. Some data were missing due to practical and/or technical issues: imagery evaluation data (*n* = 1), expected pain rating (pre-intervention*, n* = 1), or heart rate and skin conductance data (*n* = 2).

## Appendix S6: Additional analyses

Correlation of experienced pain with other outcomes Study 1. Post hoc partial correlation analyses in the response imagery condition indicated a significant association of post-intervention experienced pain with concentration on the image (*r*(32) = .549, *p* = .001), indicating that participants who were more concentrated during the imagery exercise, experienced more pain during the post-intervention CPT. Post-intervention experienced pain was not significantly associated with the other imagery evaluation variables, psychological or physiological responses.

Correlation of experienced pain with other outcomes Study 2. Post hoc partial correlation analyses in the response imagery conditions indicated a significant association of post-intervention experienced pain with thinking about the image during the CPT (*r*(86) = -.295, *p* = .005) and post-intervention positive affect (*r*(86) = -.223, *p* = .036), indicating that participants who thought about the image more and/or had higher positive affect, experienced less pain during the post-intervention CPT. Post-intervention experienced pain was not significantly associated with the other imagery evaluation variables, the other psychological responses, or physiological responses.

Sensitivity analyses Study 1. When assumptions of statistical tests were violated, bootstrapped confidence intervals around the parameters, transformations of variables, and/or removing outliers did not significantly affect the results, with two exceptions. When one extreme outlier was excluded, heart rate during the post-intervention CPT was significantly lower after response imagery than after control imagery (*F*(1,72) = 4.221, *p* = .044, *η_p_^2^* = .055). Bootstrapped 95% confidence intervals around Pearson’s *r* indicated a significant association between experienced pain and thinking about the image during the post-intervention CPT (*r*(32) = -.320, 95% CI [-.629;-.058)].

When excluding three participants who had inadvertently used medication that might have affected their responses (1 participant used an analgesic, 1 ointment for eczema, and 1 antihistamine), experienced pain was not found to be significantly lower after response imagery than after control imagery, but a trend was still observed (*F*(1,71) = 3.397, *p* = .050, *η_p_^2^* = .053). Excluding these participants did not significantly affect the results of other analyses.

Sensitivity analyses Study 2. When assumptions of statistical tests were violated, bootstrapped confidence intervals around the parameters, transformations of variables, and/or removing outliers did not significantly affect the results, with one exception. The moderation of the effect of adding a verbal suggestion on experienced pain by neuroticism was non-significant when the variables were square-root transformed (*β* = 0.215, *t* = 1.323, *p* = .190).

When one participant who indicated having complaints that can be characterized as Raynaud’s phenomenon was excluded, heart rate during the post-intervention CPT was found to be significantly higher in the imagery conditions than in the *NT Contr condition* (*F*(1,127) = 4.042, *p* = .046, *η_p_^2^* = .031), and the moderation of the effect of adding a verbal suggestion on experienced pain by neuroticism was found to be non-significant when untransformed (*β* = 0.323, *t* = 1.984, *p* = .051) or square root transformed (*β* = 0.213, *t* = 1.301, *p* = .197). Excluding this participant did not significantly affect the results of other analyses.

## Table S1. Means and standard deviations for all measures in Study 1

| **Condition**  **Measure** |  | **Response imagery** |  | **Control imagery** |
| --- | --- | --- | --- | --- |
|  |  | (*n*=39) |  | (*n*=41) |
| **Pre test-session** |  |  |  |  |
| LOT-R |  | 16.6 ± 3.5 |  | 16.9 ± 3.8 |
| EPQ-RSS neuroticism |  | 3.1 ± 2.6 |  | 3.0 ± 2.9 |
|  |  |  |  |  |
| **Pre-intervention** |  |  |  |  |
| Resting heart rate |  | 72.7 ± 11.4 |  | 74.0 ± 11.5 |
| Resting skin conductance level |  | 5.2 ± 3.5 |  | 5.1 ± 2.3 |
| Resting cortisol |  | 13.1 ± 6.1 |  | 15.0 ± 9.5 |
| Resting alpha-amylase |  | 822.9 ± 750.3 |  | 823.9 ± 962.5 |
| Expected pain |  | 4.3 ± 2.0 |  | 4.5 ± 1.7 |
| Experienced pain during CPT |  | 4.3 ± 2.1 |  | 4.5 ± 2.3 |
| PANAS-PA |  | 13.3 ± 3.3 |  | 12.4 ± 3.0 |
| PANAS-NA |  | 6.0 ± 1.0 |  | 6.0 ± 1.7 |
| STAI-S |  | 32.6 ± 8.0 |  | 31.9 ± 6.8 |
| FEXpos* |  | 45.9 ± 5.0 |  | 46.0 ± 5.0 |
| FEXneg |  | 31.9 ± 6.2 |  | 33.3 ± 6.9 |
| Heart rate during CPT |  | 76.5 ± 14.3 |  | 76.8 ± 12.1 |
| Skin conductance during CPT |  | 7.3 ± 3.2 |  | 7.5 ± 2.2 |
| Cortisol 10 min after CPT |  | 12.3 ± 4.7 |  | 14.2 ± 8.2 |
| Cortisol 20 min after CPT |  | 13.0 ± 5.7 |  | 13.7 ± 7.9 |
| Alpha-amylase 10 min after CPT |  | 742.5 ± 565.1 |  | 782.4 ± 734.1 |
| Alpha-amylase 20 min after CPT |  | 972.2 ± 1151.2 |  | 701.8 ± 652.9 |
|  |  |  |  |  |
| **Post-intervention** |  |  |  |  |
| Concentration on image |  | 63.9 ± 17.9 |  | 60.7 ± 15.7 |
| Quality visualization |  | 66.6 ± 14.1 |  | 64.3 ± 17.2 |
| Valence of image |  | 75.4 ± 16.1 |  | 66.4 ± 18.4 |
| Thinking about image during CPT |  | 56.3 ± 28.6 |  | 16.9 ± 24.9 |
| Expected pain |  | 4.8 ± 2.2 |  | 5.8 ± 2.2 |
| Experienced pain during CPT |  | 4.1 ± 2.1 |  | 4.7 ± 2.3 |
| PANAS-PA |  | 12.3 ± 3.6 |  | 11.2 ± 3.5 |
| PANAS-NA |  | 5.5 ± 1.2 |  | 5.2 ± 0.6 |
| STAI-S |  | 34.0 ± 8.3 |  | 33.8 ± 7.5 |
| FEXpos ^a^ |  | 46.2 ± 4.7 |  | 45.4 ± 4.7 |
| FEXneg |  | 32.5 ± 6.7 |  | 32.5 ± 6.3 |
| Heart rate during CPT |  | 71.4 ± 12.7 |  | 73.4 ± 10.7 |
| Skin conductance during CPT |  | 6.1 ± 3.2 |  | 6.4 ± 2.4 |
| Cortisol 10 min after CPT |  | 10.4 ± 4.0 |  | 11.4 ± 5.4 |
| Cortisol 20 min after CPT |  | 9.7 ± 3.4 |  | 10.8 ± 5.0 |
| Alpha-amylase 10 min after CPT |  | 913.9 ± 740.8 |  | 911.5 ± 860.6 |
| Alpha-amylase 20 min after CPT |  | 1049.4 ± 782.5 |  | 1161.4 ± 1377.4 |

*Note*. Means and standard deviations (*M* ± *SD*) are presented for all available data. The data used for analyses sometimes differ due to list-wise deletions in the case of missing values (see statistical analyses section Study 1 of main text). See Methods section of main text for more information on the measures.

^a^ excluding FEX item 4, to obtain a satisfactory Cronbach’s alpha for this scale.

## Table S2. Means and standard deviations for all measures in Study 2

| **Condition**  **Measure** |  | **Response imagery with verbal suggestion** |  | **Response imagery** |  | **No treatment control** |
| --- | --- | --- | --- | --- | --- | --- |
|  |  | (*n*=47) |  | (*n*=45) |  | (*n*=43) |
|  |  |  |  |  |  |  |
| **Pre test-session** |  |  |  |  |  |  |
| LOT-R |  | 16.6 ± 3.1 |  | 16.7 ± 3.4 |  | 16.3 ± 3.1 |
| EPQ-RSS neuroticism |  | 2.4 ± 2.0 |  | 3.4 ± 2.4 |  | 3.1 ± 2.7 |
| PCS |  | 9.3 ± 6.9 |  | 10.5 ± 7.8 |  | 11.3 ± 6.7 |
|  |  |  |  |  |  |  |
| **Pre-intervention** |  |  |  |  |  |  |
| Resting heart rate |  | 77.9 ± 10.5 |  | 75.7 ± 8.0 |  | 76.6 ± 11.0 |
| Resting skin conductance level |  | 4.3 ± 2.4 |  | 4.3 ± 2.3 |  | 4.1 ± 2.5 |
| Expected pain before CPT |  | 4.5 ± 1.7 |  | 4.2 ± 1.6 |  | 4.3 ± 1.6 |
| Pain anxiety before CPT |  | 1.1 ± 1.3 |  | 1.1 ± 1.3 |  | 1.3 ± 1.4 |
| Experienced pain during CPT |  | 4.8 ± 2.2 |  | 4.9 ± 2.0 |  | 4.7 ± 2.3 |
| Expected pain after CPT |  | 5.4 ± 1.9 |  | 5.2 ± 1.8 |  | 5.0 ± 2.4 |
| Pain anxiety after CPT |  | 1.1 ± 1.3 |  | 1.5 ± 1.8 |  | 1.3 ± 1.7 |
| PANAS-PA |  | 13.4 ± 3.7 |  | 12.2 ± 2.9 |  | 12.3 ± 3.3 |
| PANAS-NA |  | 6.1 ± 1.7 |  | 6.2 ± 1.9 |  | 6.4 ± 1.4 |
| STAI-S |  | 32.8 ± 7.9 |  | 35.0 ± 9.0 |  | 35.1 ± 10.0 |
| FEXpos |  | 54.7 ± 5.3 |  | 53.9 ± 6.1 |  | 53.1 ± 7.4 |
| FEXneg |  | 27.8 ± 7.7 |  | 27.8 ± 7.9 |  | 28.9 ± 8.2 |
| Heart rate during CPT |  | 80.0 ± 10.6 |  | 77.7 ± 9.1 |  | 80.2 ± 10.2 |
| Skin conductance during CPT |  | 6.3 ± 2.5 |  | 7.1 ± 2.4 |  | 6.6 ± 2.7 |
|  |  |  |  |  |  |  |
| **Post-intervention** |  |  |  |  |  |  |
| Concentration on image |  | 68.6 ± 14.3 |  | 69.3 ± 12.9 |  | - |
| Quality visualization |  | 68.5 ± 15.2 |  | 70.7 ± 14.7 |  | - |
| Valence of image |  | 78.0 ± 18.7 |  | 81.5 ± 18.0 |  | - |
| Thinking about image during CPT |  | 76.1 ± 18.7 |  | 69.3 ± 21.6 |  | - |
| Expected pain |  | 3.8 ± 1.9 |  | 4.3 ± 1.9 |  | 4.8 ± 2.3 |
| Pain anxiety |  | 0.9 ± 1.1 |  | 1.1 ± 1.5 |  | 1.5 ± 1.8 |
| Experienced pain during CPT |  | 3.9 ± 2.2 |  | 4.3 ± 1.9 |  | 4.8 ± 2.3 |
| PANAS-PA |  | 13.0 ± 3.6 |  | 12.2 ± 3.4 |  | 11.4 ± 3.2 |
| PANAS-NA |  | 5.3 ± 0.9 |  | 5.4 ± 0.7 |  | 5.5 ± 0.9 |
| STAI-S |  | 30.6 ± 6.8 |  | 33.7 ± 7.9 |  | 32.3 ± 9.0 |
| FEXpos |  | 55.8 ± 5.7 |  | 54.6 ± 5.8 |  | 53.2 ± 7.9 |
| FEXneg |  | 27.7 ± 8.1 |  | 26.9 ± 8.1 |  | 28.1 ± 8.4 |
| Heart rate during CPT |  | 79.4 ± 11.1 |  | 76.3 ± 8.5 |  | 76.9 ± 9.5 |
| Skin conductance during CPT |  | 5.6 ± 1.8 |  | 6.5 ± 2.3 |  | 6.5 ± 2.7 |

*Note*. Means and standard deviations (*M* ± *SD*) are presented for all available data. The data used for analyses sometimes differ due to list-wise deletions in the case of missing values (see statistical analyses section Study 2 of main text). See Methods section of main text for more information on the measures.

screening, demographics, psychological characteristics

**CPT**

incl. pain & physiological assessments

**Response imagery**

**CPT**

incl. pain & physiological assessments

**Control imagery**

psychological questionnaires, physiological assessments

psychological & exit questionnaires, debriefing

filler task

or

## Figure S1. Flow diagram showing the experimental procedures of Study 1 in chronological order

CPT = cold pressor test.

screening, demographics, psychological characteristics

**CPT**

incl. pain & physiological assessments

**Response imagery + Verbal suggestion**

**CPT**

incl. pain & physiological assessments

**Response imagery**

psychological questionnaires, physiological assessments

psychological & exit questionnaires, debriefing

filler task

or

**No treatment**

or

**Figure S2. Flow diagram showing the experimental procedures of Study 2 in chronological order**

CPT = cold pressor test.

## References Supporting information

Hanssen M.M., Peters M.L., Vlaeyen J.W.S., Meevissen Y.M.C., Vancleef L.M.G. (2013). Optimism lowers pain: Evidence of the causal status and underlying mechanisms. Pain 154, 53-58.

Kercher K. (1992). Assessing subjective well-being in the old-old - the panas as a measure of orthogonal dimensions of positive and negative affect. Res Aging 14, 131-168.

Kirschbaum C. and Hellhammer D.H. (1994). Salivary cortisol in psychoneuroendocrine research - recent developments and applications. Psychoneuroendocrinology 19, 313-333.

Kirschbaum C. and Hellhammer D.H. (2000). Salivary cortisol. In Encyclopedia of stress. pp. 379-383.

Marteau T.M. and Bekker H. (1992). The development of a six-item short-form of the state scale of the spielberger state-trait anxiety inventory (stai). Br J Clin Psychol 31, 301-306.

Peeters F.P.M.L., Ponds R.W.H.M., Vermeeren M.T.G. (1996). Affectiviteit en zeltbeoordeling van depressie en angst. Tijdschr Psychiatr 38, 240-250.

Peters M.L., Flink I.K., Boersma K., Linton S.J. (2010). Manipulating optimism: Can imagining a best possible self be used to increase positive future expectancies? J Posit Psychol 5, 204-211.

Rohleder N. and Nater U.M. (2009). Determinants of salivary alpha-amylase in humans and methodological considerations. Psychoneuroendocrinology 34, 469-485.

Sanderman R., Arrindell W.A., Ranchor A.V., Eysenck H.J., Eysenck S.B.G. (1995). Het meten van persoonlijkheidskenmerken met de eysenck personality questionnaire (epq): Een handleiding (Groningen: Noordelijk Centrum voor Gezondheidsvraagstukken).

Scheier M.F., Carver C.S., Bridges M.W. (1994). Distinguishing optimism from neuroticism (and trait anxiety, self-mastery, and self-esteem): A reevaluation of the life orientation test. J Pers Soc Psychol 67, 1063-1078.

Sullivan M.J.L., Bishop S.R., Pivik J. (1995). The pain catastrophizing scale: Development and validation. Psychol Assess 7, 524-532.

van der Ploeg H.M. (1982). De zelf-beoordelings vragenlijst (stai-dy). Tijdschr Psychiatr 24, 576-588.
